# Level of physical activity and aerobic capacity associate with quality of life in patients with temporal lobe epilepsy

**DOI:** 10.1371/journal.pone.0181505

**Published:** 2017-07-19

**Authors:** Nathalia Volpato, Juliana Kobashigawa, Clarissa Lin Yasuda, Simoni Thiemi Kishimoto, Paula Teixeira Fernandes, Fernando Cendes

**Affiliations:** 1 Neuroimaging Laboratory, Hospital de Clínicas, Rua Vital Brasil, University of Campinas, UNICAMP, Cidade Universitária Zeferino Vaz, Campinas, SP, Brazil; 2 Physical Education College; University of Campinas–UNICAMP, Cidade Universitária Érico Veríssimo, Campinas, SP, Brazil; Universitiy of Modena and Reggio Emilia, ITALY

## Abstract

Epilepsy is more than seizures and includes a high risk of comorbidities and psychological disorders, leading to poor quality of life (QOL). Earlier studies have showed a sedentary lifestyle in people with epilepsy (PWE), which could contribute to poorer health and psychological problems. The purpose of the present study was to compare habits of physical activity (PA), aerobic capacity, and QOL between PWE and healthy controls in order to identify the necessity of intervention of habits and information on physical exercise (PE) and to better understand the importance of PE for PWE. The study included 38 patients with temporal lobe epilepsy and 20 normal controls. Both groups answered the WHOQOL-Bref, which assesses the level of QOL, and IPAQ to evaluate the level of PA. In addition, they were submitted to a treadmill maximal cardiopulmonary effort test to identify physical capacity. The continuous variables were compared between groups by t-test and a general linear model, and the frequencies were compared by Chi-Square test through SPSS software. There was no difference in the level of PA between groups by questionnaire evaluation. However, there were significant differences in overall QOL, physical health, and level of PA in relation to work and physical capacity between groups; controls demonstrated better scores than PWE. Controls presented better physical capacity than PWE by cardiopulmonary effort test. According to intra-group analyses, PWE who were physically active had better QOL than inactive PWE. The study concluded that questionnaires about PE may not be the best instrument of evaluation, as demonstrated by the discrepancy of results compared to the validated objective cardiopulmonary evaluation of level of PA and physical capacity in this study.

## 1. Introduction

Epilepsy is a chronic disease with repercussions extending beyond the negative impact of recurrent seizures. Patients usually present cognitive dysfunction [1], in addition to side effects from anti-epileptic drugs (AEDs) [2].

Overall, epilepsy has been strongly associated with psychosocial impairment, including high rates of anxiety and depression [3–5]. It is therefore not surprising that there is a suboptimal quality of life (QOL) in some of these subjects, given the complex interaction among seizures, AED effects, comorbidities, and stigma. Efforts to improve QOL in epilepsy should not focus merely on seizure control but rather approach different aspects of life, including both physical and psychosocial well-being [6].

Unfortunately, despite the well-known beneficial effects of physical activity (PA) for humans in general [7], the effects of PA in subjects with epilepsy (more specifically, subjects with pharmacoresistant epilepsy) have not been extensively studied and explored. Controversies exist about the risks of seizures during exercises, which have not proved entirely true [8–10]; on the contrary, interventional human studies have showed benefits of physical exercise (PE) for subjects with epilepsy after an exercise-training program. They showed improvement of physical capacity, general health, and psychological state [11–13].

PA is understood as any movement produced by muscular contraction, which increases the energy expenditure of the body. PA includes activities of daily life, such as walking to places for transportation, housework activities, and work. When PA is performed regularly at an ideal intensity and frequency, it is defined as PE and promotes adaptation of the organism through morphological, biochemical, and physiological changes that can lead to the development of physical performance and health benefits [7].

A significant decrease of seizure frequency was observed by Eriksen et al. [12] in 15 women with various types of epilepsy after a dancing program. Other studies have showed improvement of physical capacity and emotional status after PE interventions [12–14]. None of these studies observed increased frequency of seizures nor alteration in the metabolism of AEDs during the exercise programs.

There is scarce evidence for PE acting as a precipitating factor of seizures, as most studies are case reports [9, 14]. Unfortunately, given the nature of such studies, this topic has not yet been well clarified. Due to these controversies, earlier studies have showed that subjects with epilepsy lead an inactive lifestyle. The low level of exercises has been associated with a combination of factors, such as fear of seizures during exercise (resulting in injuries), problems with transportation, low motivation, and side effects of medication [14–17].

Temporal lobe epilepsy (TLE) is the most common and refractory epileptic syndrome in adults [18]. The impact of PE for this specific population is not clear and has not been extensively studied [14, 19]. To understand the effects of PA in TLE, we investigated physical daily activities, physical capacity, and QOL comparing with subjects without epilepsy. We hypothesized that more active subjects should present better QOL and good physical capacity, without, however, worsening seizure frequency.

The novelty of our work was the quantitative cardiopulmonary evaluation, in addition to validated questionnaires, to investigate differences and relationships among physical capacity, physical health, and QOL in a group of patients with the same form of epilepsy (TLE) compared to controls. A homogeneous group of patients removes potential differences related to the etiology of epilepsy in the variables investigated here.

## 2. Material and methods

### 2.1 Subjects

We recruited 38 patients with TLE and 20 normal controls at the epilepsy clinic of the University of Campinas. All patients with TLE had the diagnosis based on comprehensive clinical evaluation, including detailed history, general and neurological exams, serial EEGs, and MRI analysis. Patients had either MRI signs of hippocampal sclerosis (n = 32) or normal MRI (n = 6). Patients with other types of lesions were not included.

MRI signs of hippocampal sclerosis were defined as hippocampal atrophy on T1-weighted and hyperintense hippocampal signal on T2-weighted or fluid attenuated inversion recovery (FLAIR) high resolution images acquired on a 3T scanner [20].

All subjects were free from any other acute and chronic illness (including neurological disorders other than epilepsy), did not present any injury, and did not use other medications that would contraindicate or compromise the performance of intense PA. Additional exclusion criteria were previous surgery, use of pacemakers, and pregnancy.

After selection, subjects were interviewed with questionnaires and scheduled to perform a maximum-effort cardiopulmonary exam.

All individuals were required to sign a consent form approved by the Ethics Committee of the UNICAMP Medical School.

### 2.2 Study design

We evaluated both TLE and controls, investigating their history of PA, QOL (with WHOQOL-BREF) [21], level of PA (with a second questionnaire [IPAQ]) [22], and physical capacity (by testing the maximal cardiopulmonary effort).

### 2.3 Clinical outcome measures

**Quality of life** was evaluated by the WHOQOL-BREF questionnaire. The questionnaire comprises 26 questions, which are divided into four domains: physical health, psychological health, social relationships, and environment. The answers are based on a Likert scale (1 to 5, with higher scores representing better QOL) [21].

**Level of physical activity** was evaluated with the IPAQ (International Physical Activity Questionnaire), which evaluates total physical activities performed during daily life. It is divided into five sections: PA at work, PA as means of transportation, leisure and recreational PA, and the time spent sitting [22]. The questionnaire provides four levels of PA: very active, active, irregularly active, inactive. However, given the reduced number of subjects, we combined some levels into two categories: active (from the combination of very active and active) and inactive (irregularly active and inactive). Therefore, patients and controls were subdivided into physically active and inactive.

#### Maximal effort cardiopulmonary exam on treadmill

A graded protocol on a treadmill (T2100, General Electric, Waukesha, WI, USA) was used to determine the maximum oxygen consumption (VO_2max_). The initial speed for all volunteers was 4 km/h for two minutes; it was increased by 0.5Km/h every 30 seconds with a constant slope at 1%. The test was interrupted when the subject failed to keep walking or running. We therefore initiated the calm-down step, comprising two minutes at 3.5Km/h and two minutes sitting. During the process, subjects were monitored with both an electrocardiogram and a gas analyzer (Oxycon Pro, Erich Jaeger GmbH, Hoechberg, Germany), which evaluates oxygen consumption as well as production of CO_2_. The cardiopulmonary effort test was performed in the same period of the day (early afternoon) after a light snack for all individuals, therefore controlling for the last meal intake.

Aerobic power was expressed as the VO_2max_, considering the average of values obtained the last 30 seconds of the cardiorespiratory evaluation. To confirm the VO_2max_, at least two of the following three criteria must be observed: (1) plateau in VO_2_ (no variation or little variation in VO_2_ (<2.1 mL.kg-1.min-1) despite the increase of the exercise intensity); (2) Ratio of respiratory changes greater than 1.10; (3) Heart rate (HR) greater than 90% of the maximum predicted for age [23].

**Anthropometric data** were assessed with body mass index (BMI). Individual classification was determined according to the World Health Organization (WHO) [24] anthropometric classification.

**Seizure frequency** was computed based on patient’s diaries over six months prior to the evaluation. Since all patients were pharmacoresistant, except two, we divided them into two groups according to seizure frequency: (1) below and (2) above three seizures per month. We evaluate the difference between these two extremes of seizure frequency in relation to the QOL, level of physical activity, physical capacity, and BMI.

### 2.4 Statistical analyses

All clinical variables, anthropometric data, and demographic data were analyzed with SPSS statistic software (SPSS, Chicago, IL, USA).

Normal distribution was examined with the Kolmogorov–Smirnov test. Differences between groups (as well as patient subgroups) were evaluated with t-test. Distribution of categorical variables was analyzed by Chi-Square test or Fisher’s exact test. We used a general linear model (GLM) to analyze QOL and cardiopulmonary test results, covarying for the level of PA, using patients and controls as contrast groups. The level of significance adopted was a p value equal to or less than 0.05.

## 3. Results

The 38 TLE patients had a mean age of 43 years (standard deviation [SD] ± 10.16 years, ranging from 23 to 59 years, 14 men and 24 women), and the 20 controls had a mean age of 46 years (SD ± 8.43 years, ranging from 27 to 58 years, 7 men and 13 women). Controls and patients were balanced for age (p = 0.44) and gender (p = 0.3), and they all shared the same social condition (Table 1).

**Table 1:** Characteristics of sample.

	TLEG (n = 38)	CG (n = 20)	p value
Age (range)	43 ± 10.16(23 to 59 years)	46 ± 8.43(27 to 58 years)	0.44
Gender	14 males	7 males	0.3

TLE: temporal lobe epilepsy group

Controls: normal control group. p<0.05

According to the IPAQ scores, 27 TLE patients were considered physically active and had a mean age of 44 years (± 8.01 years, 11 men and 16 women), and 11 patients were inactive and had a mean age of 40 years (± 14.08 years, 3 men and 8 women). There was no difference in seizure frequency and no difference in AED load between active and inactive patients (Table 2).

**Table 2:** Characteristics of TLE-Active and TLE-Inactive.

	TLE-Active (n = 27)	TLE-Inactive (n = 11)	p values
Mean Age ± SD (range)	44 ± 8,1 (23 to 59)	40 ± 14,08 (23 to 59)	0.38
Gender	11 males, 16 females	3 males, 8 females	0.48
Lesional or MRI negative	21 lesional, 6 MRI Negative	11 lesional	0.15
Side of TLE	13 lTLE, 7 rTLE, 1 BilTLE, 6 NegTLE	4 lTLE, 5 rTLE, 2 BilTLE	0.12
Mean age at seizure onset ± SD (range)	13.34 ± 9.5 (6 months to 40 years)	10.22 ± 9.35 (6 months to 30 years)	0.36
Seizure frequency *	2.56 per month	4.36 per month	0.16
AEDS	Monotherapy (n = 8)	Monotherapy (n = 4)	0.7

TLE-Active: temporal lobe epilepsy group that is considered active; TLE-Inactive: group of patients who are considered inactive; lELT: left ELT; rELT: right ELT; BilELT: Bilateral ELT; NegELT: negative MRI ELTAEDS: anti-epileptic drugs.

* All patients were pharmacoresistant, except two (one in each group).

There were 16 physically active controls, who had a mean age of 47 years (± 8.54 years; 5 men and 11 women), and four inactive controls, who had a mean age of 42 years (± 7.67, two men and two women) (Table 2).

### 3.1 Quality of life data

**Fig 1:**
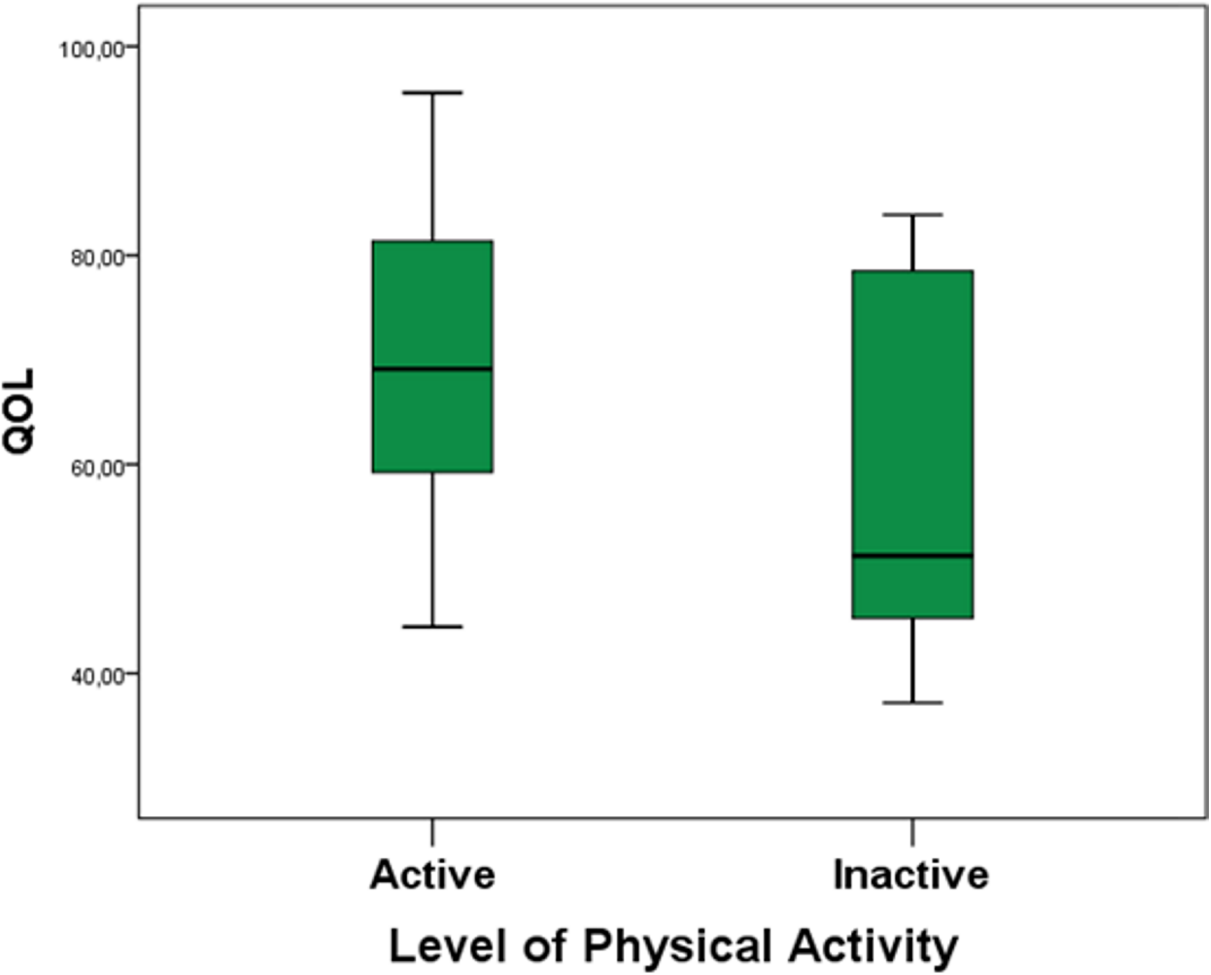
Comparison of QOL within TLE, between TLE-Active and TLE-Inactive. **Active presented higher values for QOL.** QOL: quality of life; TLE-Active: temporal lobe epilepsy group that is considered active; TLE-Inactive: group of patients who are considered inactive; p = statistically significant value.

We observed a difference in QOL between groups when we covariate these data with the level of PA. Therefore, we could observe the influence of PA on QOL (p = 0.02) and physical health (p = 0.01), with higher scores in controls. In addition, in the subsequent analysis of QOL within sub-groups, TLE-Active presented better QOL than TLE-Inactive (p = 0.04) (Fig 1).

We found no significant differences between controls and TLE regarding average QOL and their domains (physical health, psychological health, social relationships, and environment) (S1 File). There were no differences between males and females in QOL scores, neither relationship with clinical data such as epilepsy onset, side of TLE and seizures frequency, AED (Table 2 and S1 File).

### 3.2 Daily physical activity profile

**Fig 2:**
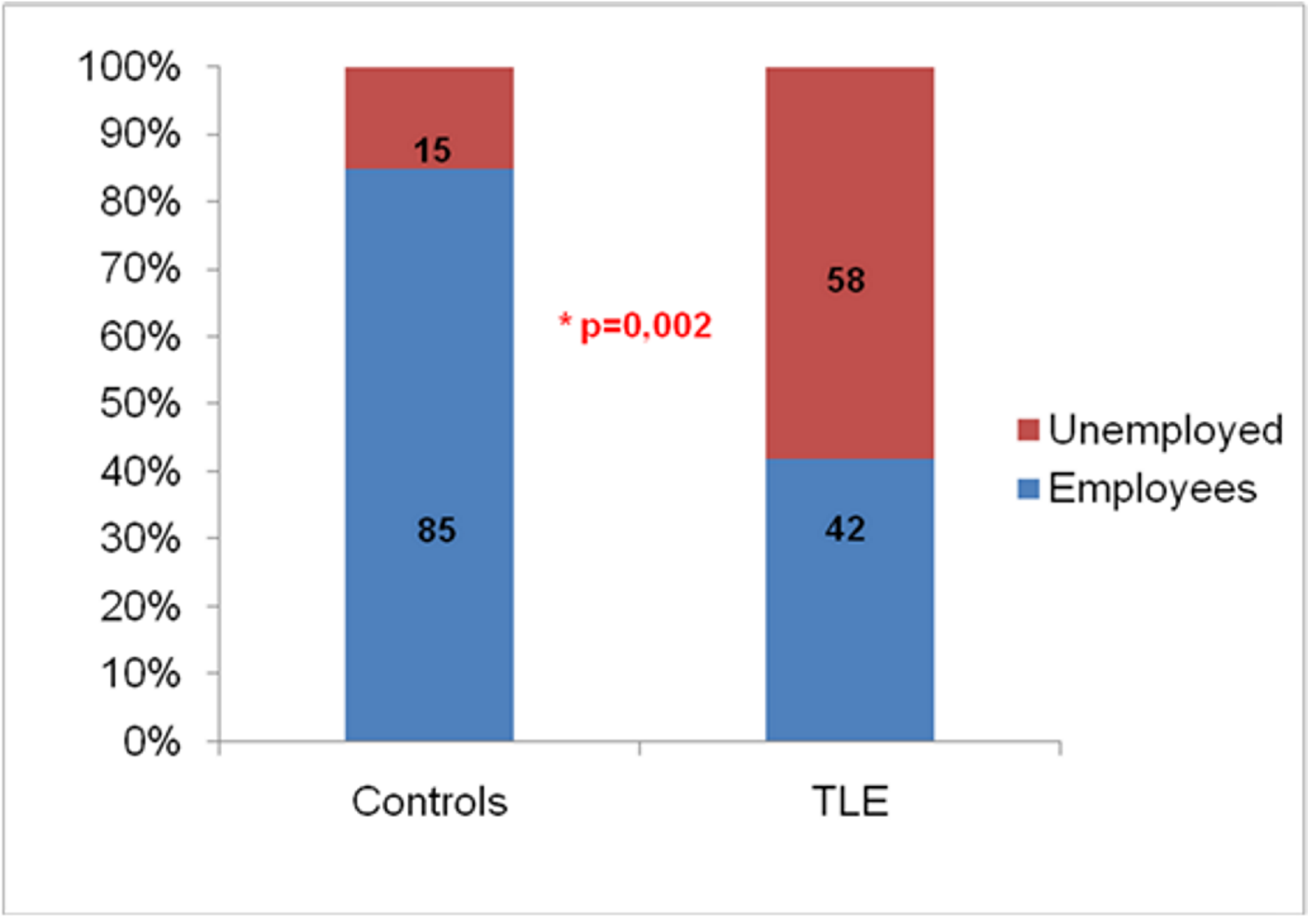
Comparison of employment frequencies between control and TLE. TLE: temporal lobe epilepsy group; Controls: normal control group.

Comparing the PA habits profile, we did not observe differences in the level of PA between controls and TLE (p = 0.54). However, controls were more often employed (85%) than TLE (42%) (p = 0.002) (Fig 2), which probably explains why the TLE group had more people performing leisure PA (23% versus 5% in controls; p = 0.14).

### 3.3 Physical capacity data

**Fig 3:**
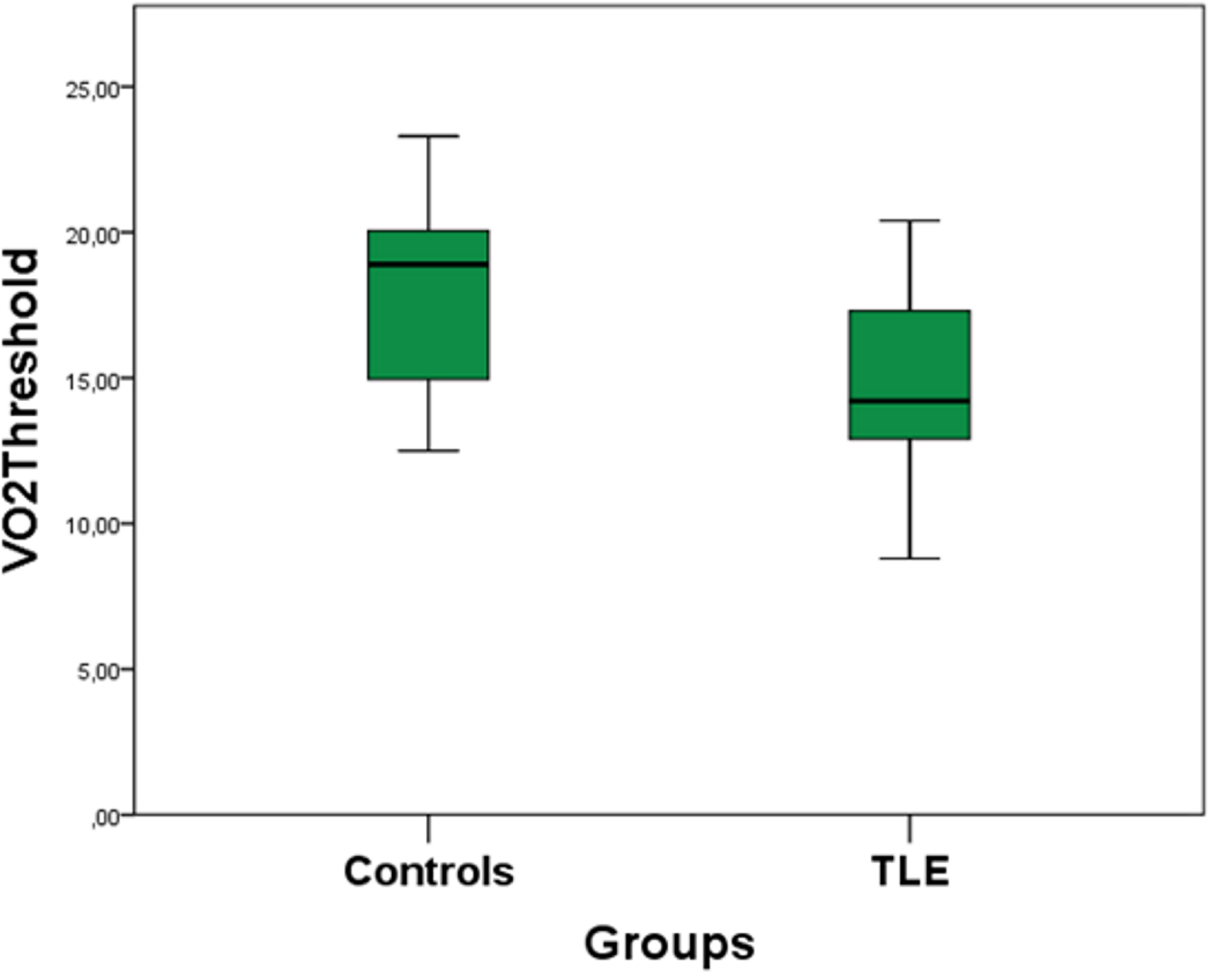
Comparison of physical capacity data between TLE and controls. **Controls presented higher value of VO**_**2threshold**_ **than TLE.** TLE: temporal lobe epilepsy group; Controls: control group (without epilepsy); VO_2threshold_: value of O_2_ in the threshold between aerobic and anaerobic metabolism; p = statistically significant value.

**Fig 4:**
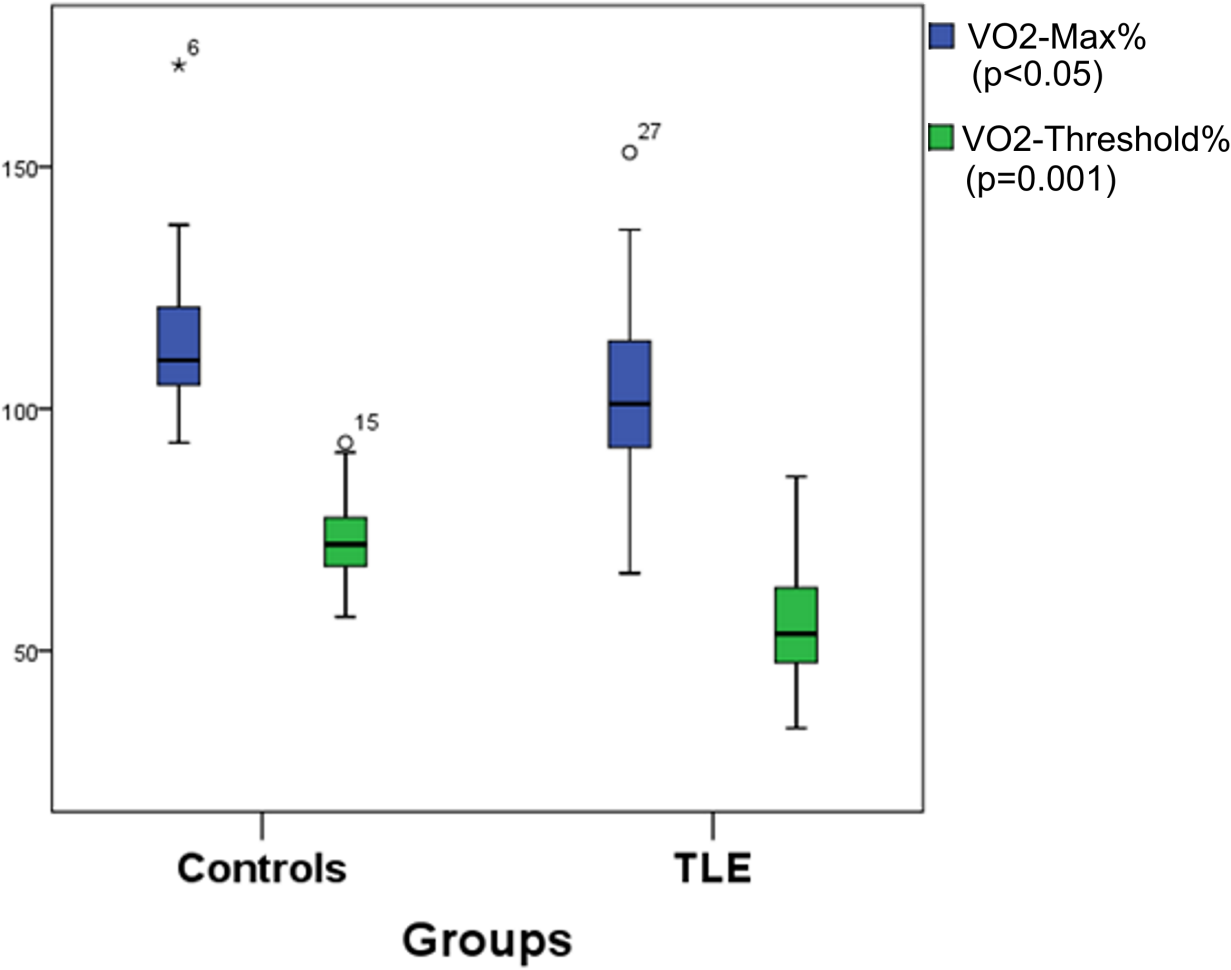
Comparison of physical capacity data between TLE and controls. **Controls presented higher value of VO**_**2maxpercent**_ **and VO**_**2thresholdpercent**_ **than TLE.** TLE: temporal lobe epilepsy group; Controls: control group (without epilepsy); VO_2maxpercent_: percent to age of max value of O_2_ reached in maximal cardiopulmonary effort; VO_2thresholdpercent_: percent to age of O_2_ reached in the threshold between aerobic and anaerobic metabolism.

We observed significant differences in physical capacity, measured by percent to age of O_2_ values reached in maximal cardiopulmonary effort (VO_2 maxpercent_) and values of O_2_ in the threshold between aerobic and anaerobic metabolism (VO_2threshold_) between controls and TLE. The control group presented better physical capacity than TLE (VO_2maxpercent_ p = 0.05; VO_2threshold_ p = 0.001 and percent to age of O_2_ reached in the threshold between aerobic and anaerobic metabolism [VO_2thresholdpercent_], p = 0.001) (Fig 3 and Fig 4).

There were no differences between males and females in physical capacity (S1 File) neither significant relationships between clinical data such as epilepsy onset, side of TLE and seizures frequency and physical activity and physical capacity (S1 File).

Considering all recruited subjects (patients and controls), we did not identify a single person with excellent or good physical capacity. All subjects were classified with regular or poor aerobic capacity. Not a single person with TLE presented seizures during or after the cardiopulmonary effort exam.

There was no significant difference in anthropometric data among groups (S1 File).

### 3.4 Seizure frequency

**Fig 5:**
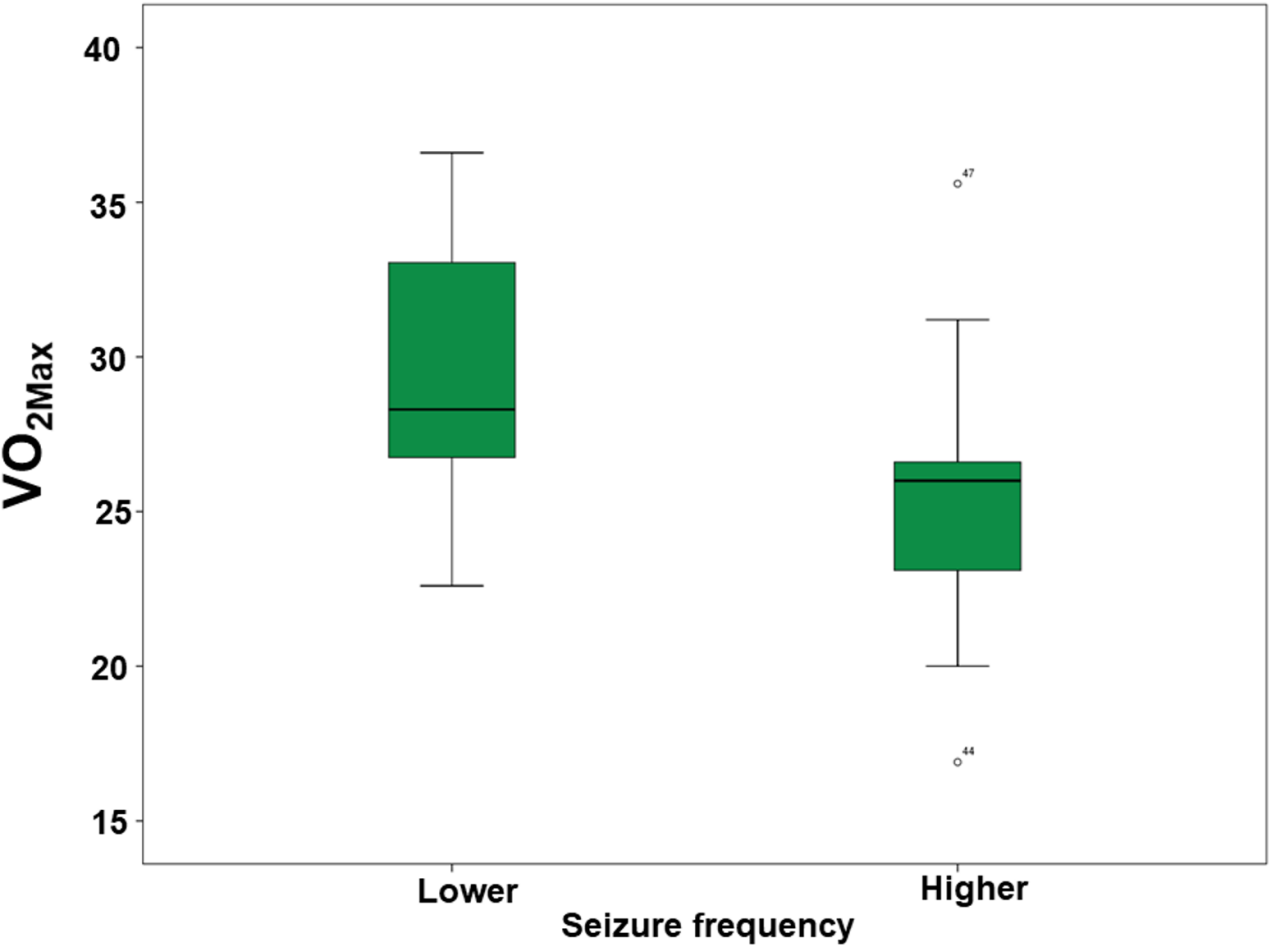
Comparison of physical capacity data between patients with lower seizure frequency and patients with higher seizure frequency. **Patients with lower seizure frequency had higher values of VO**_**2max**_ **than patients with higher seizure frequency.** VO_2max_: percent to age of maximal value of O_2_ reached in maximal cardiopulmonary effort; Lower seizure frequency: group of patients with less than three seizures per month; Higher seizure frequency: group of patients with more than three seizures per month.

There was no difference in the QOL, level of physical activity and BMI scores among the two seizure frequency groups as described above (S1 File). Physical capacity, however, was better in the group with lower seizure frequency (less than three seizures per month) (Fig 5).

## 4. Discussion

Different from most studies in people with epilepsy (PWE) about PA that used only questionnaires, we included additional quantitative state-of-the-art cardiopulmonary evaluation to compare TLE patients and controls from the same social environment. Our results showed that the level of PA between our controls and patients did not differ per questionnaires; however, controls had better physical capacity than PWE according to the quantitative cardiopulmonary evaluation. In addition, TLE patients who were physically active had better QOL than those considered inactive.

### 4.1 Physical activity habit data

In contrast to previous studies [15, 16, 25], we observed no significant difference in the overall level of PA between groups. This corroborates with population-based studies that found an equivalent level of PA and leisure PA between PWE and the general population [26, 27]. However, both groups did not present good or great capacity, just regular or poor, showing that both groups need to be encouraged to practice PE.

However, most our patients reported being unemployed, thus not practicing PA related to work. On the other hand, most of the controls were employed. These data confirm previous studies that showed higher levels of unemployment for PWE compared to the general population [15, 28]. This fact may explain why the groups did not present a significant difference in the level of overall daily PA; while TLE patients practiced more leisure physical activity, controls presented more activity related to work.

### 4.2 Quality of life data

Our initial analyses showed equivalent QOL for both groups. However, when co-variating for level of PA in a GLM, we found that PWE had poorer QOL than controls.

Previous studies have showed that PWE usually have a lower level of QOL (mainly due to the frequency of seizures, the stigma at work, and the comorbidities related to the disease) [29, 30]; however, no previous study has controlled QOL for PA.

Interestingly, we demonstrated a better level of QOL for TLE-Active compared to TLE-Inactive patients. So far, no previous studies have evaluated the interaction between PA and QOL specifically in TLE; however, it is well known that QOL in individuals with epilepsy is affected by mood disorders, rather than only AED treatment and social factors [5].

This finding is consistent with data obtained by Roth et al. [31], who observed that the level of depression is lower among PWE who exercise regularly. Intervention and population studies [11–13] have showed that PE can be a complementary treatment strategy to improve the mood state of PWE; thus, our data corroborate with these studies and highlight the importance of PE to improve QOL, which is affected in this population [5, 31].

### 4.3 Physical capacity data

Previous studies using questionnaires showed a lower level of PA in PWE [15, 16]. We confirmed this finding using a quantitative cardiopulmonary test, showing that controls had better physical capacity than PWE.

It is interesting that, even though patients have a higher level of leisure PA, they have worse overall physical capacity than controls. This can be explained partly by significant differences in employability. This suggests that work has a great influence on individuals’ physical capacity, and patients who practice leisure PA are not practicing at an ideal exercise intensity to promote adequate physical health adaptations. According to these data, we can infer that PWE need to practice more leisure PA at an ideal intensity to reach the physical capacity, ideally under professional supervision.

The VO_2_ value achieved in the cardiopulmonary effort exam is a determinant factor of aerobic capacity. This value is used to evaluate the risk of cardiovascular and pulmonary comorbidities, and it is a predictor of general health [32]. Our study found that PWE who were active had better QOL, physical health, and VO_2_ values (physical capacity) compared to patients who did not practice exercises.

Experimental animal models of epilepsy have showed PE mechanisms that can promote the improvement of several aspects related to epilepsy, such as delayed kindling model development, improvement of spatial memory, increased brain-derived neurotrophic factor and hilo fibers of dentate gyrus in the hippocampus, and decreased frequency and intensity of seizures and spikes in the EEG during PA and after a PE training program [33–37]. This explains some neurological PE adaptations that can lead to clinical improvements in animals, which need to be better understood in humans. Our data showed that patients with better physical capacity had less seizure frequency, thus, indicating a relationship that need to be explore in further studies, since we cannot define a causal relationship due to the cross-sectional design of the present study.

## 5. Conclusion

Our study showed no overall difference in the level of PA between controls and the general population using validated questionnaires; however, we found that PWE had poorer physical capacity using a state-of-the-art cardiopulmonary effort exam. The objectively measured physical capacity seems to be better discriminating variable than IPAQ score.

There were differences in QOL and physical health between groups, with better scores in controls. In addition, patients who were active had better QOL than patients who were physically inactive.

There are probable benefits in performing cardiopulmonary evaluation to prescribe the adequate intensity of a PE program for PWE. Whether this will improve QOL and seizure control needs to be investigated in future trials.

## Supporting information

S1 File Additional statistical analyses. Descriptive statistics and output statistics for quality of life, level of physical capacity, and physical capacity group analyses.
(DOCX)

## Abbreviations

TLE: Temporal lobe epilepsy
PWE: People with epilepsy
Controls: Control group
QOL: Quality of life
