# Supplementary material for: Level of physical activity and aerobic capacity associate with quality of life in patients with temporal lobe epilepsy
Source: PLoS One. 2017 Jul 19;12(7):e0181505. doi: 10.1371/journal.pone.0181505 (PMC5517033; doi:10.1371/journal.pone.0181505)
Supplement: S1 File — Descriptive statistics and output statistics for quality of life, level of physical capacity, and physical capacity group analyses. (DOCX) [file pone.0181505.s001.docx]

**Supplemental data**

**Additional statistical analyses**

**Quality of life group analyses**

Comparisons of means between QOL, sub-items of QOLIE-31 and Gender.

| Source | Dependent Variable | p |
| --- | --- | --- |
|  | QOL | 0.27 |
| Gender | Social Relationships | 0.01 |
|  | Physical Health | 0.61 |
|  | Psychological Health | 0.18 |
|  | Social Relationships | 0.26 |

Footnote: QOL: quality of life. p<0.05

Correlation between QOL and sub-items of QOLIE-31with epilepsy onset.

|  | | QOL | Physical  Health | Psychological  Health | Social  Relationships |
| --- | --- | --- | --- | --- | --- |
| EO | Pearson Correlation | -0.06 | -0.13 | 0.05 | 0.01 |
|  | Sig. (2-tailed) | 0.71 | 0.43 | 0.73 | 0.91 |
|  | N | 38 | 38 | 38 | 38 |

Footnote: QOL: quality of life; EO: epilepsy onset. p<0.05

| Comparisons of means between QOL and EGEGE E TLE side | | | |
| --- | --- | --- | --- |
|  | | Value Label | N |
| TLE Side | 1,00 | LTLE | 17 |
|  | 2,00 | RTLE | 12 |
|  | 3,00 | BIL | 3 |
|  | 4,00 | NEG | 6 |

Footnote: NEG: LTLE: left temporal lobe epilepsy, RTLE: right temporal lobe epilepsy, BIL: bilateral.

**Means of QOL and sub-items of QOLIE-31 according to TLE subgroups.**

| Dependent Variable | TLE-Side | Mean |
| --- | --- | --- |
|  |  |  |
| QOL | LTLE | 67.99 |
|  | RTLE | 63.35 |
|  | BIL | 68.97 |
|  | NEG | 69.04 |
| Physical Health | LTLE | 72.79 |
|  | RTLE | 67.75 |
|  | BIL | 80.66 |
|  | NEG | 74.29 |
| Psychological Health | LTLE | 62.91 |
|  | RTLE | 56.14 |
|  | BIL | 66.58 |
|  | NEG | 67.25 |
| Social Relationships | LTLE | 67.98 |
|  | RTLE | 64.47 |
|  | BIL | 69.33 |
|  | NEG | 64.91 |

Footnote: NEG: LTLE: left temporal lobe epilepsy, RTLE: right temporal lobe epilepsy, BIL: bilateral.

**P values of comparisons of means of QOL and sub-items of QOLIE-31 between TLE side.**

| Source | Dependent Variable | p |
| --- | --- | --- |
| TLE Side | Physical Health | 0.64 |
|  | Psychological Health | 0.70 |
|  | Social Relationships | 0.97 |

Footnote: QOL: quality of life. p<0.05

**Comparisons of means between QOL and sub-items of QOLIE-31 with seizure frequency.**

| Source | Dependent Variable |  |  |  | p |
| --- | --- | --- | --- | --- | --- |
| sz_freq | QOL |  |  |  | 0.63 |
|  | Physical Health |  |  |  | 0.79 |
|  | Psychological Health |  |  |  | 0.74 |
|  | Social Relationships |  |  |  | 0.34 |

Footnote: sz_freq: seizure frequency (lower frequency or higher frequency), QOL: quality of life. p<0.05

**Comparisons of means between QOL and sub-items o QOLIE-31 with anti-epileptic drugs**

| Source | Dependent Variable | p |
| --- | --- | --- |
| AED | QOL | 0.68 |
|  | Physical Health | 0.4 |
|  | Psychological Health | 0.67 |
|  | Social Relationships | 0.16 |

Footnote: AED: ant-epileptic drug (monotherapy or polytherapy), QOL: quality of life. p<0.05

**Level of physical capacity group analyses**

| **Frequency between gender and level of physical activity** | | | | |
| --- | --- | --- | --- | --- |
|  | | Gender | | Total |
|  |  | Female | Male |  |
| Physical Activity | Active | 16 | 11 | 27 |
|  | Inactive | 8 | 3 | 11 |
| Total | | 24 | 14 | 38 |

| **p values for comparisons of proportions of gender according to level of physical activity** | | | | | |
| --- | --- | --- | --- | --- | --- |
|  | Value | df | Asymp. Sig. (2-sided) | Exact Sig. (2-sided) | Exact Sig. (1-sided) |
| Pearson Chi-Square | 0.60^a^ | 1 | 0.43 |  |  |
| Continuity Correction^b^ | 0.16 | 1 | 0.68 |  |  |
| Likelihood Ratio | 0.62 | 1 | 0.42 |  |  |
| Fisher's Exact Test |  |  |  | 0.48 | 0.34 |
| Linear-by-Linear Association | 0.59 | 1 | 0.44 |  |  |
| N of Valid Cases | 38 |  |  |  |  |

| **Comparisons of means between epilepsy onset and level of physical activity** | | | | | |
| --- | --- | --- | --- | --- | --- |
|  | Physical Activity | N | Mean | Std. Deviation | p |
| EO | Active | 27 | 13.34 | 9.50 | 0.36 |
|  | Inactive | 11 | 10.22 | 9.35 |  |

Footnote: EO: epilepsy onset. p<0.05

| **TLE subgroups versus level of physical activity** | | | | | | |
| --- | --- | --- | --- | --- | --- | --- |
|  | | | | | | |
|  | | TLE Side | | | | Total |
|  |  | LTLE | RTLE | BIL | NEG |  |
| Physical Activity | Active | 13 | 7 | 1 | 6 | 27 |
|  | Inactive | 4 | 5 | 2 | 0 | 11 |
| Total | | 17 | 12 | 3 | 6 | 38 |

Footnote: NEG: LTLE: left temporal lobe epilepsy, RTLE: right temporal lobe epilepsy, BIL: bilateral.

| **Chi-Square Tests** | | | |
| --- | --- | --- | --- |
|  | Value | df | Asymp. Sig. (2-sided) |
| Pearson Chi-Square | 5.70^a^ | 3 | 0.12 |
| Likelihood Ratio | 7.05 | 3 | 0.07 |
| Linear-by-Linear Association | 0.218 | 1 | 0.64 |
| N of Valid Cases | 38 |  |  |

| **Frequency of seizure frequency between level of physical activity** | | | | |
| --- | --- | --- | --- | --- |
|  | | | | |
|  | | sz_freq | | Total |
|  |  | low_freq | high_freq |  |
| Physical Activity | Active | 15 | 12 | 27 |
|  | Inactive | 5 | 6 | 11 |
| Total | | 20 | 18 | 38 |

Footnote: sz_freq: seizure frequency, Low_freq: low frequency; high_freq: high frequency.

| **p of values of comparisons between seizure frequency and level of level of physical activity** | | | | | |
| --- | --- | --- | --- | --- | --- |
|  | Value | df | Asymp. Sig. (2-sided) | Exact Sig. (2-sided) | Exact Sig. (1-sided) |
| Pearson Chi-Square | 0.32^a^ | 1 | 0.57 |  |  |
| Continuity Correction^b^ | 0.04 | 1 | 0.83 |  |  |
| Likelihood Ratio | 0.32 | 1 | 0.57 |  |  |
| Fisher's Exact Test |  |  |  | 0.72 | 0.41 |
| Linear-by-Linear Association | 0.31 | 1 | 0.57 |  |  |
| N of Valid Cases | 38 |  |  |  |  |

Footnote: p>0.05

| **Physical Activity versus AED Cross tabulation** | | | | |
| --- | --- | --- | --- | --- |
|  | | AED | | Total |
|  |  | Monotherapy | Polytherapy |  |
| Physical Activity | Active | 8 | 19 | 27 |
|  | Inactive | 4 | 7 | 11 |
| Total | | 12 | 26 | 38 |

Footnote: AED: anti-epileptic drug

| **p values for comparisons of proportions of AED monotherapy and polytherapy according to the level of level of physical activity** | | | | | |
| --- | --- | --- | --- | --- | --- |
|  | Value | df | Asymp. Sig. (2-sided) | Exact Sig. (2-sided) | Exact Sig. (1-sided) |
| Pearson Chi-Square | 0.16^a^ | 1 | 0.68 |  |  |
| Continuity Correction^b^ | 0.00 | 1 | 0.98 |  |  |
| Likelihood Ratio | 0.16 | 1 | 0.68 |  |  |
| Fisher's Exact Test |  |  |  | 0.71 | 0.48 |
| Linear-by-Linear Association | 0.16 | 1 | 0.68 |  |  |
| N of Valid Cases | 38 |  |  |  |  |

Footnote: AED: anti-epileptic drugs. P>0.05.

**Physical capacity group analyses**

| **Tests of Between-Subjects Effects** | | | | | | |
| --- | --- | --- | --- | --- | --- | --- |
| Gender | VO_2Max_ | 76,342 | 1 | 76,342 | 3,894 | 0.05 |
|  | VO_2MaxPercent_ | 521,382 | 1 | 521,382 | 1,604 | 0.21 |
|  | VO_2Threshold_ | 34,807 | 1 | 34,807 | 4,356 | 0.04 |
|  | VO_2ThresholdPercent_ | 365,687 | 1 | 365,687 | 2,037 | 0.16 |

| **Correlations** | | | | | |
| --- | --- | --- | --- | --- | --- |
|  | | VO_2Max_ | VO_2MaxPercent_ | VO_2Threshold_ | VO_2ThresholdPercent_ |
| EO | Pearson Correlation | -,106 | -,116 | ,120 | ,098 |
|  | Sig. (2-tailed) | 0.52 | 0.49 | 0.48 | 0.56 |
|  | N | 38 | 37 | 37 | 36 |

Footnote: EO: epilepsy onset.

| **Between-Subjects Factors** | | | |
| --- | --- | --- | --- |
|  | | Value Label | N |
| ELT Side | 1,00 | LTLE | 16 |
|  | 2,00 | RTLE | 12 |
|  | 3,00 | BIL | 3 |
|  | 4,00 | NEG | 5 |

| **Descriptive Statistics** | | | | |
| --- | --- | --- | --- | --- |
|  | ELT-Side | Mean | Std. Deviation | N |
| VO_2Max_ | LTLE | 26.56 | 4.26 | 16 |
|  | RTLE | 29.90 | 5.13 | 12 |
|  | BIL | 26.40 | 4.80 | 3 |
|  | NEG | 25.26 | 2.33 | 5 |
|  | Total | 27.48 | 4.60 | 36 |
| VO_2MaxPercent_ | LTLE | 99.18 | 19.40 | 16 |
|  | RTLE | 107.25 | 19.21 | 12 |
|  | BIL | 100.66 | 9.07 | 3 |
|  | NEG | 105.40 | 17.38 | 5 |
|  | Total | 102.86 | 18.18 | 36 |
| VO_2Threshold_ | LTLE | 13.45 | 2.78 | 16 |
|  | RTLE | 15.77 | 3.13 | 12 |
|  | BIL | 15.26 | 2.51 | 3 |
|  | NEG | 15.98 | 2.29 | 5 |
|  | Total | 14.73 | 2.95 | 36 |
| VO_2ThresholdPercent_ | LTLE | 50.62 | 12.54 | 16 |
|  | RTLE | 58.00 | 11.59 | 12 |
|  | BIL | 58.66 | 4.04 | 3 |
|  | NEG | 68.40 | 18.03 | 5 |
|  | Total | 56.22 | 13.59 | 36 |

| **Tests of Between-Subjects Effects** | | | | | | |  |
| --- | --- | --- | --- | --- | --- | --- | --- |
| Source | Dependent Variable | Type III Sum of Squares | df | Mean Square | F | Sig. | |
| TLE Side | VO_2Max_ | 112.18 | 3 | 37.9 | 1.89 | 0.15 |  |
|  | VO_2MaxPercent_ | 493.751 | 3 | 164.584 | 0.47 | 0.70 |  |
|  | VO_2Threshold_ | 47.74 | 3 | 15.91 | 1.96 | 0.13 |  |
|  | VO_2ThresholdPercent_ | 1298.60 | 3 | 432.86 | 2.67 | 0.06 |  |

| **Between-Subjects Factors** | | | |
| --- | --- | --- | --- |
|  | | Value Label | N |
| sz_freq | 9,00 | low_freq | 19 |
|  | 10,00 | high_freq | 17 |

| **Tests of Between-Subjects Effects** | | | | | | |
| --- | --- | --- | --- | --- | --- | --- |
| Source | Dependent Variable | Type III Sum of Squares | df | Mean Square | F | Sig. |
| sz_freq | VO_2Max_ | 171,825 | 1 | 171,82 | 10,231 | 0.003 |
|  | VO_2MaxPercent_ | 149,618 | 1 | 149,61 | ,445 | 0.50 |
|  | VO_2Threshold_ | 18,032 | 1 | 18,032 | 2,125 | 0.15 |
|  | VO_2ThresholdPercent_ | 3,040 | 1 | 3,04 | ,016 | 0.90 |

| **AED monotherapy versus polytherapy** | | | |
| --- | --- | --- | --- |
|  | | Value Label | N |
| AED | 1,00 | Monotherapy | 12 |
|  | 2,00 | Polytherapy | 24 |

| **Tests of Between-Subjects Effects** | | | | | | |
| --- | --- | --- | --- | --- | --- | --- |
| Source | Dependent Variable | Type III Sum of Squares | df | Mean Square | F | Sig. |
| AED | VO_2Max_ | 3,167 | 1 | 3,167 | ,146 | 0.70 |
|  | VO_2MaxPercent_ | 133,389 | 1 | 133,389 | ,396 | 0.53 |
|  | VO_2Threshold_ | 2,683 | 1 | 2,683 | ,300 | 0.58 |
|  | VO_2ThresholdPercent_ | 224,014 | 1 | 224,014 | 1,219 | 0.27 |
